# Crystal structure of 1-hy­droxy-2,2,6,6-tetra­methyl­piperidin-1-ium tri­fluoro­methane­sulfonate

**DOI:** 10.1107/S2056989015020897

**Published:** 2015-11-07

**Authors:** Christian Godemann, Anke Spannenberg, Torsten Beweries

**Affiliations:** a Leibniz-Institut für Katalyse e.V. an der Universität Rostock, Albert-Einstein-Strasse 29a, 18059 Rostock, Germany

**Keywords:** crystal structure, TEMPO, ammonium salt, triflate, hydrogen bonding

## Abstract

In the cation of the title salt, C_9_H_20_NO^+^·CF_3_O_3_S^−^, the six-membered heterocyclic ring displays a chair conformation. In the crystal, centrosymmetric pairs of cations and anions are linked by N—H⋯O and O—H⋯O hydrogen bonds to form rings with a *R*_4_^4^(14) graph-set motif.

## Related literature   

For mol­ecular structures and discussions of related compounds, see: Jaitner & Wurst (1997 ▸); Spirk *et al.* (2010 ▸); Ananchenko *et al.* (2006 ▸); Percino *et al.* (2016 ▸). For the mol­ecular structure of the neutral TEMPO-H compound, see: Mader *et al.* (2007 ▸); Giffin *et al.* (2011 ▸).
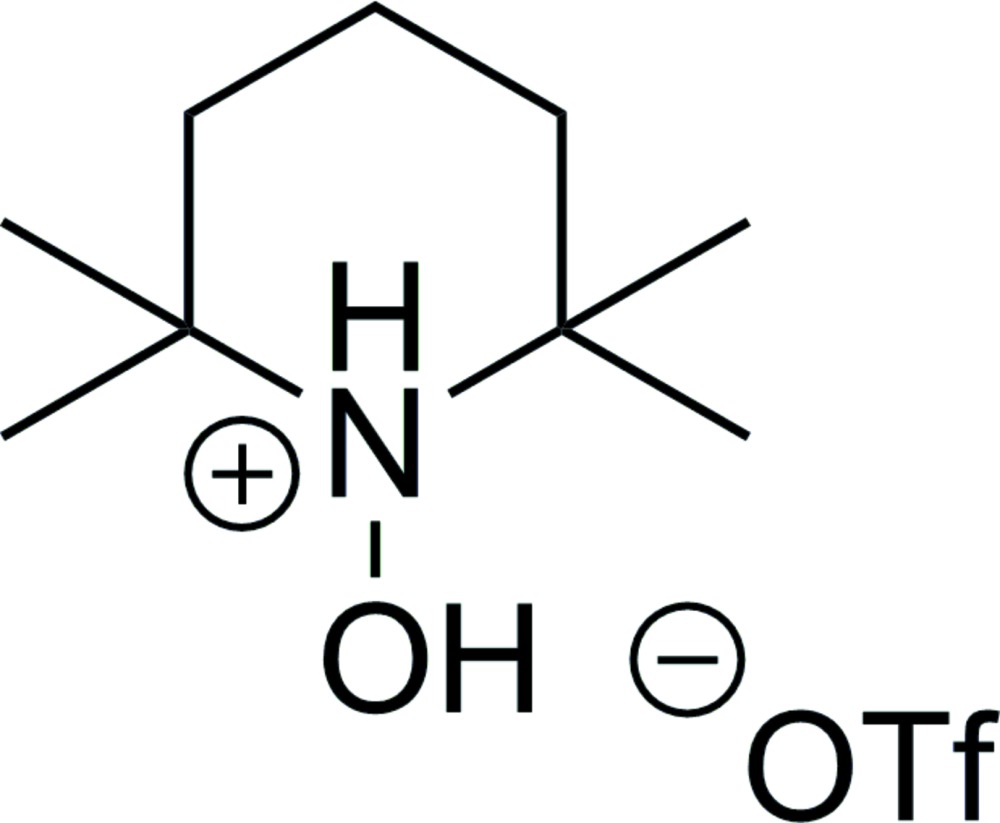


## Experimental   

### Crystal data  

C_9_H_20_NO^+^·CF_3_O_3_S^−^
*M* *_r_* = 307.33
Triclinic, 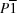

*a* = 8.2824 (2) Å
*b* = 8.7656 (2) Å
*c* = 10.5703 (3) Å
α = 79.5417 (7)°
β = 76.5159 (7)°
γ = 75.5022 (6)°
*V* = 716.28 (3) Å^3^
*Z* = 2
Mo *K*α radiation
μ = 0.27 mm^−1^
*T* = 150 K
0.55 × 0.38 × 0.34 mm

### Data collection  

Bruker APEXII CCD diffractometer
Absorption correction: multi-scan (*SADABS*; Bruker, 2014 ▸) *T*_min_ = 0.83, *T*_max_ = 0.86
22933 measured reflections
3452 independent reflections
3039 reflections with *I* > 2σ(*I*)
*R*_int_ = 0.020

### Refinement  

*R*[*F* ^2^ > 2σ(*F* ^2^)] = 0.034
*wR*(*F* ^2^) = 0.094
*S* = 1.05
3452 reflections
184 parameters
H atoms treated by a mixture of independent and constrained refinement
Δρ_max_ = 0.32 e Å^−3^
Δρ_min_ = −0.28 e Å^−3^

### 

Data collection: *APEX2* (Bruker, 2014 ▸); cell refinement: *SAINT* (Bruker, 2013 ▸); data reduction: *SAINT*; program(s) used to solve structure: *SHELXS97* (Sheldrick, 2008 ▸); program(s) used to refine structure: *SHELXL2014* (Sheldrick, 2015 ▸); molecular graphics: *SHELXTL* (Sheldrick, 2008 ▸); software used to prepare material for publication: *SHELXL2014*.

## Supplementary Material

Crystal structure: contains datablock(s) I, New_Global_Publ_Block. DOI: 10.1107/S2056989015020897/rz5176sup1.cif

Structure factors: contains datablock(s) I. DOI: 10.1107/S2056989015020897/rz5176Isup2.hkl

Supporting information file. DOI: 10.1107/S2056989015020897/rz5176Isup3.cml

**Fig. 1.**
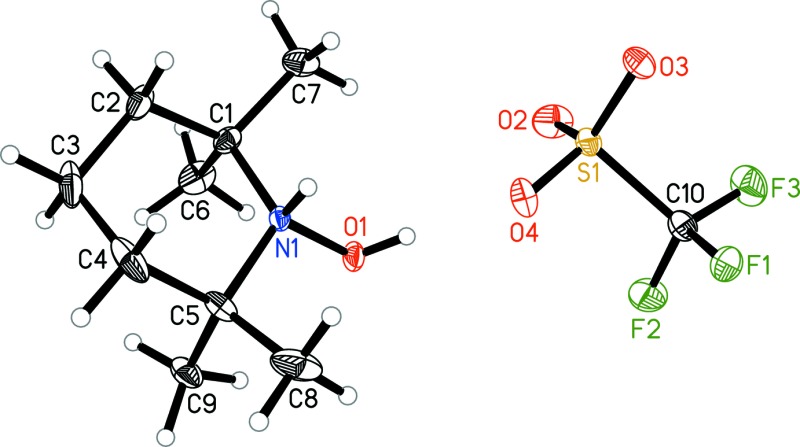
The molecular structure of the title compound with displacement ellipsoids drawn at the 30% probability level.

. DOI: 10.1107/S2056989015020897/rz5176fig1.tif
The mol­ecular structure of the title compound with displacement ellipsoids drawn at the 30% probability level.

**Fig. 2.**
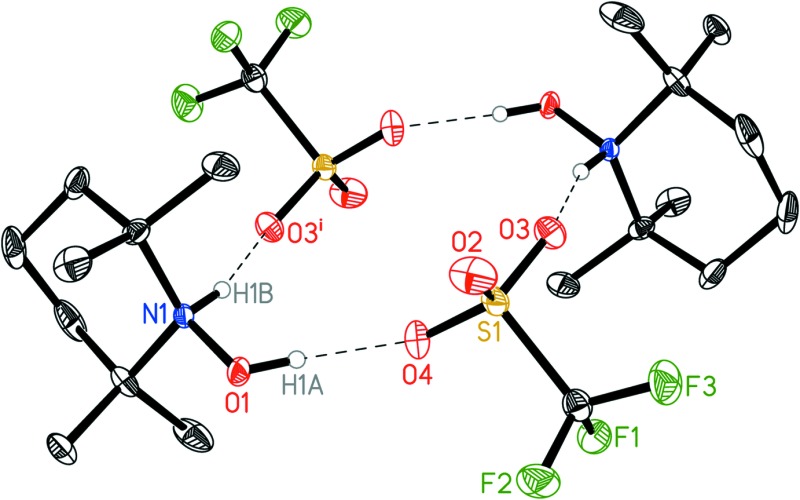
The hydrogen-bonding network (dashed lines) linking centrosymmetric pairs of cations and anions in the title compound. C-bound hydrogen atoms are omitted for clarity. Displacement ellipsoids are drawn at the 30% probability level.

. DOI: 10.1107/S2056989015020897/rz5176fig2.tif
The hydrogen-bonding network (dashed lines) linking centrosymmetric pairs of cations and anions in the title compound. C-bound hydrogen atoms are omitted for clarity. Displacement ellipsoids are drawn at the 30% probability level.

CCDC reference: 1435030

Additional supporting information:  crystallographic information; 3D view; checkCIF report

## Figures and Tables

**Table 1:** Hydrogen-bond geometry (Å, °)

*D*—H⋯*A*	*D*—H	H⋯*A*	*D*⋯*A*	*D*—H⋯*A*
O1—H1*A*⋯O4	0.84 (2)	1.78 (2)	2.6163 (14)	177 (2)
N1—H1*B*⋯O3^i^	0.875 (16)	1.991 (16)	2.8385 (14)	163.0 (14)

Symmetry code: (i) 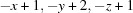.

## supplementary crystallographic information

### S1. Synthesis and crystallization

An equimolar mixture of the titanocene(IV) triflate complex [(SiMe_2_C_5_Me_4_)_2_Ti(H_2_O)(OH)(OTf)] (Godemann & Beweries, unpublished results) and 2,2,6,6-tetra­methyl-1-hy­droxy­piperidine (TEMPO-H) in toluene was cooled to -78°C. After two weeks, the formation of colourless crystals of the title compound could be observed on slow evaporation of the solvent. Alternatively, layering a toluene solution of the same titanocene compound and TEMPO-H with *n*-hexane also resulted in the formation of colourless crystals of the title compound.

### S2. Refinement

The H1A and H1B atoms were found from a difference Fourier map and refined freely. All other H atoms were placed in idealized positions with d(C—H) = 0.99 Å (CH_2_), 0.98 Å (CH_3_) and refined using a riding model with *U*_iso_(H) fixed at 1.2 *U*_eq_(C) for CH_2_ and 1.5 *U*_eq_(C) for CH_3_. A rotating model was used for the methyl groups.

### Crystal data

**Table:**

C_9_H_20_NO^+^·CF_3_O_3_S^−^	*Z* = 2
*M**_r_* = 307.33	*F*(000) = 324
Triclinic, *P*1	*D*_x_ = 1.425 Mg m^−^^3^
*a* = 8.2824 (2) Å	Mo *K*α radiation, λ = 0.71073 Å
*b* = 8.7656 (2) Å	Cell parameters from 9968 reflections
*c* = 10.5703 (3) Å	θ = 2.6–28.9°
α = 79.5417 (7)°	µ = 0.27 mm^−^^1^
β = 76.5159 (7)°	*T* = 150 K
γ = 75.5022 (6)°	Prism, colourless
*V* = 716.28 (3) Å^3^	0.55 × 0.38 × 0.34 mm

### Data collection

**Table:**

Bruker APEXII CCD diffractometer	3452 independent reflections
Radiation source: fine-focus sealed tube	3039 reflections with *I* > 2σ(*I*)
Detector resolution: 8.3333 pixels mm^-1^	*R*_int_ = 0.020
φ and ω scans	θ_max_ = 28.0°, θ_min_ = 2.0°
Absorption correction: multi-scan (*SADABS*; Bruker, 2014)	*h* = −10→10
*T*_min_ = 0.83, *T*_max_ = 0.86	*k* = −11→11
22933 measured reflections	*l* = −13→13

### Refinement

**Table:**

Refinement on *F*^2^	0 restraints
Least-squares matrix: full	Hydrogen site location: mixed
*R*[*F*^2^ > 2σ(*F*^2^)] = 0.034	H atoms treated by a mixture of independent and constrained refinement
*wR*(*F*^2^) = 0.094	*w* = 1/[σ^2^(*F*_o_^2^) + (0.0476*P*)^2^ + 0.2597*P*] where *P* = (*F*_o_^2^ + 2*F*_c_^2^)/3
*S* = 1.05	(Δ/σ)_max_ = 0.001
3452 reflections	Δρ_max_ = 0.32 e Å^−^^3^
184 parameters	Δρ_min_ = −0.28 e Å^−^^3^

### Special details

**Table:**

Geometry. All e.s.d.'s (except the e.s.d. in the dihedral angle between two l.s. planes) are estimated using the full covariance matrix. The cell e.s.d.'s are taken into account individually in the estimation of e.s.d.'s in distances, angles and torsion angles; correlations between e.s.d.'s in cell parameters are only used when they are defined by crystal symmetry. An approximate (isotropic) treatment of cell e.s.d.'s is used for estimating e.s.d.'s involving l.s. planes.

### Fractional atomic coordinates and isotropic or equivalent isotropic displacement parameters (Å^2^)

**Table:**

	*x*	*y*	*z*	*U*_iso_*/*U*_eq_	
C1	0.96872 (15)	0.80108 (15)	0.28534 (12)	0.0251 (3)	
C2	0.96590 (19)	0.86192 (17)	0.14106 (13)	0.0371 (3)	
H2A	0.8640	0.9487	0.1352	0.045*	
H2B	1.0673	0.9072	0.1019	0.045*	
C3	0.9637 (2)	0.7356 (2)	0.06137 (14)	0.0503 (5)	
H3A	1.0691	0.6517	0.0611	0.060*	
H3B	0.9595	0.7834	−0.0305	0.060*	
C4	0.8101 (2)	0.66311 (19)	0.11958 (15)	0.0456 (4)	
H4A	0.8100	0.5820	0.0655	0.055*	
H4B	0.7057	0.7472	0.1151	0.055*	
C5	0.80421 (17)	0.58576 (15)	0.26184 (13)	0.0287 (3)	
C6	1.13968 (18)	0.69450 (19)	0.30494 (17)	0.0396 (3)	
H6A	1.2234	0.7600	0.2937	0.059*	
H6B	1.1782	0.6194	0.2402	0.059*	
H6C	1.1273	0.6356	0.3936	0.059*	
C7	0.92379 (19)	0.93950 (17)	0.36579 (16)	0.0360 (3)	
H7A	0.9328	0.8983	0.4571	0.054*	
H7B	0.8072	0.9985	0.3625	0.054*	
H7C	1.0026	1.0105	0.3294	0.054*	
C8	0.6305 (2)	0.5497 (2)	0.3250 (2)	0.0511 (5)	
H8A	0.6018	0.4827	0.2724	0.077*	
H8B	0.5446	0.6494	0.3293	0.077*	
H8C	0.6334	0.4938	0.4138	0.077*	
C9	0.94213 (19)	0.43502 (16)	0.27559 (15)	0.0343 (3)	
H9A	0.9560	0.4085	0.3671	0.051*	
H9B	1.0498	0.4523	0.2192	0.051*	
H9C	0.9093	0.3473	0.2493	0.051*	
C10	0.48030 (17)	0.77783 (16)	0.86989 (13)	0.0301 (3)	
F1	0.32733 (11)	0.76163 (11)	0.86237 (9)	0.0403 (2)	
F2	0.57731 (13)	0.63247 (11)	0.88758 (10)	0.0488 (3)	
F3	0.46049 (15)	0.84651 (13)	0.97606 (9)	0.0522 (3)	
N1	0.82282 (12)	0.71302 (11)	0.33670 (9)	0.0180 (2)	
O1	0.82554 (13)	0.64554 (11)	0.46877 (8)	0.0285 (2)	
O2	0.74038 (13)	0.88719 (14)	0.74267 (14)	0.0495 (3)	
O3	0.45998 (13)	1.04716 (11)	0.72367 (10)	0.0352 (2)	
O4	0.56749 (14)	0.80767 (14)	0.61969 (10)	0.0425 (3)	
S1	0.57433 (4)	0.89340 (4)	0.72262 (3)	0.02752 (10)	
H1A	0.745 (3)	0.700 (2)	0.516 (2)	0.050 (5)*	
H1B	0.730 (2)	0.7873 (18)	0.3356 (15)	0.025 (4)*	

### Atomic displacement parameters (Å^2^)

**Table:**

	*U*^11^	*U*^22^	*U*^33^	*U*^12^	*U*^13^	*U*^23^
C1	0.0187 (6)	0.0249 (6)	0.0285 (6)	−0.0058 (4)	−0.0015 (5)	0.0019 (5)
C2	0.0327 (7)	0.0331 (7)	0.0270 (6)	0.0054 (6)	0.0074 (5)	0.0086 (5)
C3	0.0644 (11)	0.0464 (9)	0.0167 (6)	0.0215 (8)	0.0011 (6)	−0.0030 (6)
C4	0.0617 (10)	0.0393 (8)	0.0359 (8)	0.0188 (7)	−0.0286 (7)	−0.0221 (6)
C5	0.0290 (6)	0.0228 (6)	0.0368 (7)	0.0021 (5)	−0.0114 (5)	−0.0145 (5)
C6	0.0203 (6)	0.0402 (8)	0.0531 (9)	−0.0038 (6)	−0.0086 (6)	0.0050 (7)
C7	0.0354 (7)	0.0303 (7)	0.0484 (8)	−0.0133 (6)	−0.0121 (6)	−0.0068 (6)
C8	0.0323 (8)	0.0330 (8)	0.0957 (15)	−0.0088 (6)	−0.0148 (8)	−0.0230 (8)
C9	0.0393 (8)	0.0223 (6)	0.0381 (7)	0.0055 (5)	−0.0090 (6)	−0.0112 (5)
C10	0.0286 (7)	0.0293 (6)	0.0315 (7)	−0.0007 (5)	−0.0078 (5)	−0.0068 (5)
F1	0.0280 (4)	0.0431 (5)	0.0489 (5)	−0.0120 (4)	0.0008 (4)	−0.0084 (4)
F2	0.0473 (6)	0.0309 (5)	0.0588 (6)	0.0034 (4)	−0.0127 (4)	0.0043 (4)
F3	0.0693 (7)	0.0580 (6)	0.0319 (5)	−0.0098 (5)	−0.0128 (4)	−0.0148 (4)
N1	0.0177 (5)	0.0180 (4)	0.0160 (4)	−0.0009 (4)	−0.0013 (3)	−0.0031 (3)
O1	0.0368 (5)	0.0252 (4)	0.0151 (4)	0.0002 (4)	0.0018 (4)	−0.0004 (3)
O2	0.0206 (5)	0.0412 (6)	0.0850 (9)	−0.0062 (4)	−0.0104 (5)	−0.0052 (6)
O3	0.0290 (5)	0.0264 (5)	0.0432 (6)	0.0058 (4)	−0.0055 (4)	−0.0057 (4)
O4	0.0435 (6)	0.0459 (6)	0.0332 (5)	−0.0042 (5)	0.0055 (4)	−0.0172 (5)
S1	0.01852 (16)	0.02389 (17)	0.03577 (18)	0.00056 (11)	−0.00008 (12)	−0.00683 (12)

### Geometric parameters (Å, º)

**Table:**

C1—C6	1.5247 (18)	C7—H7A	0.9800
C1—C2	1.5251 (18)	C7—H7B	0.9800
C1—C7	1.5279 (19)	C7—H7C	0.9800
C1—N1	1.5362 (15)	C8—H8A	0.9800
C2—C3	1.514 (2)	C8—H8B	0.9800
C2—H2A	0.9900	C8—H8C	0.9800
C2—H2B	0.9900	C9—H9A	0.9800
C3—C4	1.516 (3)	C9—H9B	0.9800
C3—H3A	0.9900	C9—H9C	0.9800
C3—H3B	0.9900	C10—F3	1.3262 (16)
C4—C5	1.528 (2)	C10—F1	1.3314 (16)
C4—H4A	0.9900	C10—F2	1.3323 (16)
C4—H4B	0.9900	C10—S1	1.8202 (15)
C5—C8	1.522 (2)	N1—O1	1.4168 (12)
C5—C9	1.5244 (17)	N1—H1B	0.875 (16)
C5—N1	1.5354 (15)	O1—H1A	0.84 (2)
C6—H6A	0.9800	O2—S1	1.4260 (11)
C6—H6B	0.9800	O3—S1	1.4406 (9)
C6—H6C	0.9800	O4—S1	1.4486 (11)
			
C6—C1—C2	112.20 (11)	C1—C7—H7B	109.5
C6—C1—C7	110.16 (12)	H7A—C7—H7B	109.5
C2—C1—C7	110.70 (11)	C1—C7—H7C	109.5
C6—C1—N1	111.77 (10)	H7A—C7—H7C	109.5
C2—C1—N1	106.75 (11)	H7B—C7—H7C	109.5
C7—C1—N1	104.98 (10)	C5—C8—H8A	109.5
C3—C2—C1	113.88 (12)	C5—C8—H8B	109.5
C3—C2—H2A	108.8	H8A—C8—H8B	109.5
C1—C2—H2A	108.8	C5—C8—H8C	109.5
C3—C2—H2B	108.8	H8A—C8—H8C	109.5
C1—C2—H2B	108.8	H8B—C8—H8C	109.5
H2A—C2—H2B	107.7	C5—C9—H9A	109.5
C2—C3—C4	109.99 (11)	C5—C9—H9B	109.5
C2—C3—H3A	109.7	H9A—C9—H9B	109.5
C4—C3—H3A	109.7	C5—C9—H9C	109.5
C2—C3—H3B	109.7	H9A—C9—H9C	109.5
C4—C3—H3B	109.7	H9B—C9—H9C	109.5
H3A—C3—H3B	108.2	F3—C10—F1	107.06 (11)
C3—C4—C5	113.93 (13)	F3—C10—F2	108.19 (12)
C3—C4—H4A	108.8	F1—C10—F2	107.48 (11)
C5—C4—H4A	108.8	F3—C10—S1	111.63 (10)
C3—C4—H4B	108.8	F1—C10—S1	111.42 (9)
C5—C4—H4B	108.8	F2—C10—S1	110.87 (10)
H4A—C4—H4B	107.7	O1—N1—C5	108.32 (9)
C8—C5—C9	109.71 (12)	O1—N1—C1	109.24 (9)
C8—C5—C4	111.57 (14)	C5—N1—C1	120.28 (9)
C9—C5—C4	112.76 (11)	O1—N1—H1B	108.1 (10)
C8—C5—N1	105.18 (11)	C5—N1—H1B	105.5 (10)
C9—C5—N1	111.54 (10)	C1—N1—H1B	104.8 (10)
C4—C5—N1	105.78 (11)	N1—O1—H1A	107.7 (13)
C1—C6—H6A	109.5	O2—S1—O3	115.62 (7)
C1—C6—H6B	109.5	O2—S1—O4	115.69 (7)
H6A—C6—H6B	109.5	O3—S1—O4	113.73 (7)
C1—C6—H6C	109.5	O2—S1—C10	103.77 (7)
H6A—C6—H6C	109.5	O3—S1—C10	103.23 (6)
H6B—C6—H6C	109.5	O4—S1—C10	102.32 (7)
C1—C7—H7A	109.5		
			
C6—C1—C2—C3	71.46 (16)	C2—C1—N1—O1	176.56 (9)
C7—C1—C2—C3	−165.02 (13)	C7—C1—N1—O1	−65.88 (12)
N1—C1—C2—C3	−51.30 (15)	C6—C1—N1—C5	−72.58 (14)
C1—C2—C3—C4	58.13 (16)	C2—C1—N1—C5	50.45 (13)
C2—C3—C4—C5	−59.31 (16)	C7—C1—N1—C5	168.01 (10)
C3—C4—C5—C8	166.79 (12)	F3—C10—S1—O2	65.11 (11)
C3—C4—C5—C9	−69.21 (15)	F1—C10—S1—O2	−175.25 (9)
C3—C4—C5—N1	52.95 (14)	F2—C10—S1—O2	−55.59 (12)
C8—C5—N1—O1	64.27 (13)	F3—C10—S1—O3	−55.88 (11)
C9—C5—N1—O1	−54.59 (13)	F1—C10—S1—O3	63.76 (10)
C4—C5—N1—O1	−177.53 (10)	F2—C10—S1—O3	−176.58 (10)
C8—C5—N1—C1	−169.19 (12)	F3—C10—S1—O4	−174.21 (10)
C9—C5—N1—C1	71.95 (14)	F1—C10—S1—O4	−54.57 (11)
C4—C5—N1—C1	−50.99 (14)	F2—C10—S1—O4	65.09 (11)
C6—C1—N1—O1	53.53 (13)		

### Hydrogen-bond geometry (Å, º)

**Table:**

*D*—H···*A*	*D*—H	H···*A*	*D*···*A*	*D*—H···*A*
O1—H1*A*···O4	0.84 (2)	1.78 (2)	2.6163 (14)	177 (2)
N1—H1*B*···O3^i^	0.875 (16)	1.991 (16)	2.8385 (14)	163.0 (14)

Symmetry code: (i) −*x*+1, −*y*+2, −*z*+1.

## References

[bb1] Ananchenko, G. S., Pojarova, M., Udachin, K. A., Leek, D. M., Coleman, A. W. & Ripmeester, J. A. (2006). *Chem. Commun.* pp. 386–388. 10.1039/b511810g 16493807

[bb2] Bruker (2013). *SAINT*. Bruker AXS Inc., Madison, Wisconsin, USA.

[bb3] Bruker (2014). *APEX2* and *SADABS*. Bruker AXS Inc., Madison, Wisconsin, USA.

[bb4] Giffin, N. A., Makramalla, M., Hendsbee, A. D., Robertson, K. N., Sherren, C., Pye, C. C., Masuda, J. D. & Clyburne, J. A. C. (2011). *Org. Biomol. Chem.* **9**, 3672–3680. 10.1039/c0ob00999g 21472176

[bb5] Jaitner, P. & Wurst, K. (1997). *Inorg. Chim. Acta*, **255**, 95–98.

[bb7] Mader, E. A., Davidson, E. R. & Mayer, J. M. (2007). *J. Am. Chem. Soc.* **129**, 5153–5166. 10.1021/ja0686918 PMC2628630 17402735

[bb6] Percino, M. J., Cerón, M., Soriano-Moro, G., Pacheco, J. A., Castro, M. E., Chapela, V. M., Bonilla-Cruz, J. & Saldivar-Guerra, E. (2016). *J. Mol. Struct.* **1103**, 254–264.

[bb8] Sheldrick, G. M. (2008). *Acta Cryst.* A**64**, 112–122. 10.1107/S0108767307043930 18156677

[bb9] Sheldrick, G. M. (2015). *Acta Cryst.* C**71**, 3–8.

[bb10] Spirk, S., Belaj, F., Madl, T. & Pietschnig, R. (2010). *Eur. J. Inorg. Chem.* pp. 289–297.

