# Supplementary figures and images for: Crystal structure of 1-hy­droxy-2,2,6,6-tetra­methyl­piperidin-1-ium tri­fluoro­methane­sulfonate
Source: Acta Crystallogr E Crystallogr Commun. 2015 Nov 7;71(Pt 12):o921. doi: 10.1107/S2056989015020897 (PMC4719881; doi:10.1107/S2056989015020897)

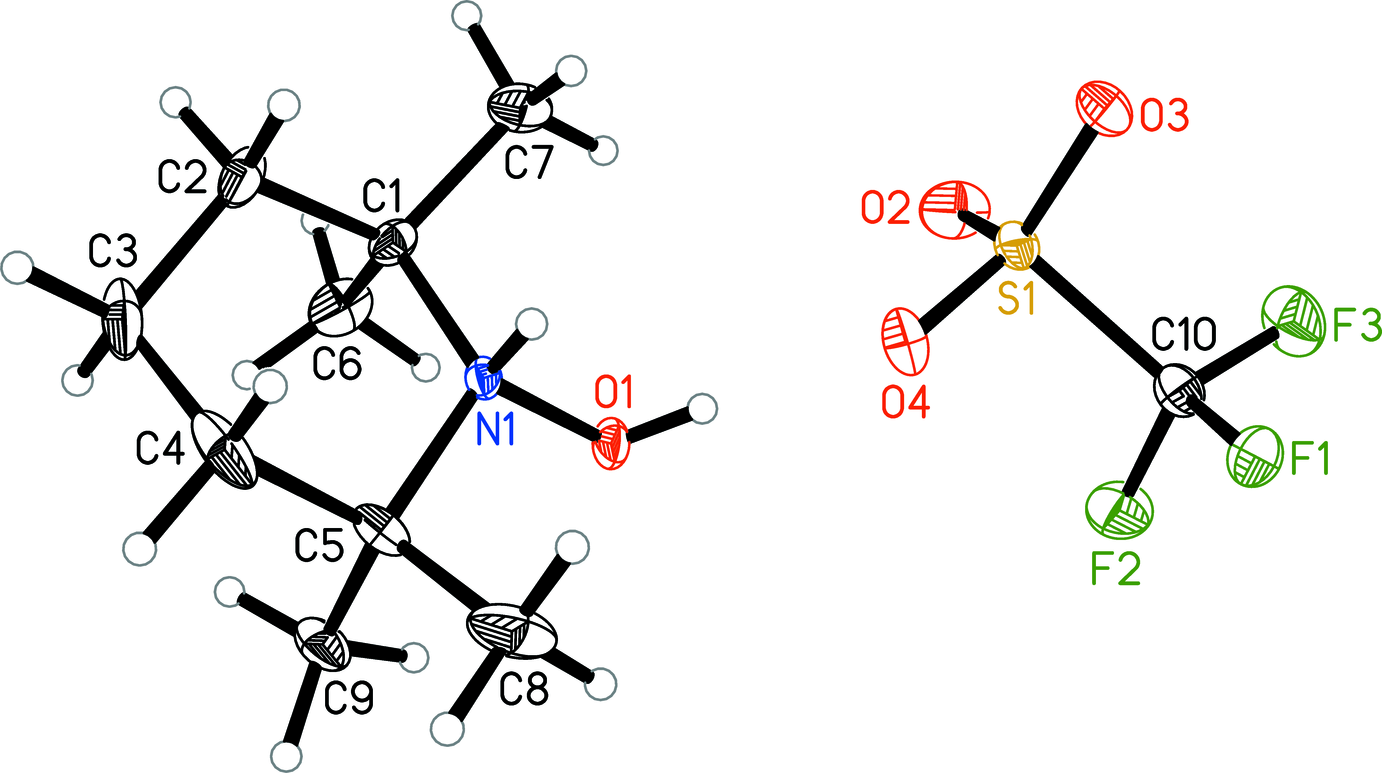

Supplement: Supplementary file 4 [file e-71-0o921-fig1.tif]

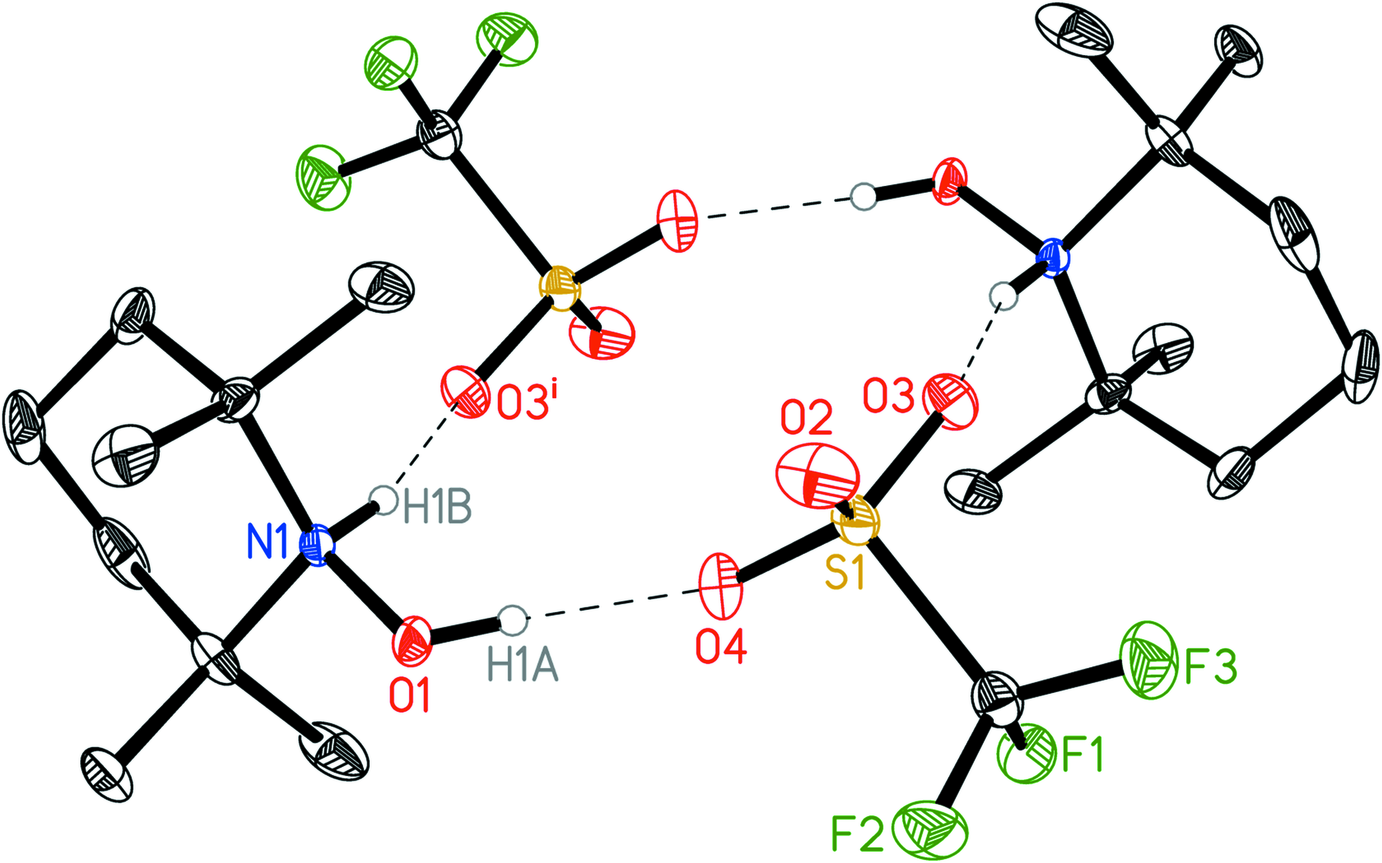

Supplement: Supplementary file 5 [file e-71-0o921-fig2.tif]
